# How Does Environmental Regulation Affect the Location of New Polluting Firms? Exploring the Agglomeration Threshold of Effective Environmental Regulation

**DOI:** 10.3390/ijerph17041279

**Published:** 2020-02-17

**Authors:** Yinhao Wu, Changhong Miao, Jianming Miao, Yan Zhang

**Affiliations:** 1 Key Research Institute of Yellow River Civilization and Sustainable Development & Collaborative Innovation Center on Yellow River Civilization of Henan Province, Henan University, Kaifeng 450001, China; 13663812426@163.com (Y.W.); Zhangyan941104@163.com (Y.Z.); 2 College of Environment and Planning, Henan University, Kaifeng 475004, China; 3 School of Economics, Henan University, Kaifeng 475004, China; miao@vip.henu.edu.cn

**Keywords:** environmental regulation, location of new polluting firms, agglomeration threshold, chemical industry, China’s Yangtze River Economic Belt

## Abstract

Some scholars have already proved the important role of agglomeration in studying how environmental regulation (ER) affects the location of polluting firms. However, further research is needed on both the mechanism and the empirical evidence. This paper reports the construction of a location database of new chemical plants in China’s Yangtze River Economic Belt (YREB), where a fixed-effects panel threshold regression model was used to explore the agglomeration threshold of effective ER. We found a single agglomeration threshold for the whole YREB region that represented the turning point of ER from excluding to attracting new chemical enterprises. Additionally, there were two agglomeration thresholds in the lower reaches. If agglomeration reached the lower threshold, the effect of ER changed from repulsion to nonsignificant attraction. Once above the upper threshold, the attraction effect became large and significant. The results for this region were consistent with the Porter hypothesis. Furthermore, there was a single agglomeration threshold in the middle reaches. When agglomeration level exceeded the threshold, the repellant effect of ER was no longer significant. In the upper reaches, we found no valid threshold and ER always exhibited a small and nonsignificant exclusion effect. The pollution haven hypothesis was more explanatory in the middle and upper reaches. In the end, some suggestions are provided to support the government to formulate differentiated environmental policies.

## 1. Introduction

The influence of environmental regulation (ER) on the location selection of new polluting firms is mainly reflected in the attraction of regions with new comparative advantages that are formed by the internalization of ER [1,2]. Stringent ER incurs additional costs and may increase barriers to entry. Conversely, ER may also stimulate innovation, enhancing the location stickiness of firms [3] and even attracting more newcomers [4]. The pollution haven hypothesis (PHH) and the Porter hypothesis (PH) suggest two operational mechanisms for the location dynamics of polluting firms. In academic circles, there is a long-standing debates over the question of which hypothesis is correct, but no consensus has been reached. Accordingly, determining the optimal institutional arrangements for pollution control remains an important unresolved public policy issue [5].

In general, the existing literature on the influence of environmental policies on the location behavior of polluting enterprises can be classified into three categories: negative effects [6,7,8], nonsignificant effects or even positive effects [9,10,11], and uncertain effects [12,13]. Specifically, Xing and Kolstad [14] found that the looser environmental policy in a destination country is, the more attractive it is to polluting industries such as the chemical industry. Li et al. [15] found that foreign chemical pharmaceutical plants in China tend to invest more in regions with loose ERs. In contrast, Levinson [9] found that the differences in US interstate ERs did not systematically affect the location behavior of most manufacturers. Similar evidence was also observed in France [16]. Ederington et al. [17] concluded that the relatively fixed industrial characteristics of polluting industries make them less sensitive to increases in environmental costs. Kikpartick and Shimamoto [10] confirmed that Japanese inward foreign direct investment (FDI) in the chemical industry and four other dirty industries occurred more often in regions with more stringent ERs. Khder and Zugravu [11] argued that in developing countries, stringent ER is more attractive to direct investment from French firms. Their explanation was that firms are forward-looking when they invest abroad. As a third perspective, according to Zhou et al. [12], only when the pollution control costs of polluting firms exceed a certain threshold value will ER have an impact on the location selection of polluting plants. That is, ER has a threshold effect on the location selection of polluting plants [13].

Shen et al. [18] summarized these variables, geographic scales, and methodologies as the reasons for the mixed results. Zheng and Shi [19] found that the types of environmental policy instruments used affect the validity of the PHH. Rezza [20] attributed the inconsistencies to the differences in variables and geographic scales. Jeppesen and Folmer [21], Jeppesen et al. [5], and List et al. [7] ascribed these mixed results to methodological issues and argued that existing paradigms may seriously underestimate the impact of ER. Although these factors partly explain the contradictions, more research on this issue is clearly needed.

Even if the factors noted above are controlled, the results obtained may still be problematic if the agglomeration effect is not well controlled in the investment equation [22]. Zeng and Zhao [23] pointed out that a common problem in these conflicting studies is that many of them have failed to fully consider the characteristics of pollution-intensive industries, namely, imperfect competition and increasing return of scale. This problem was manifested in an empirical test as neglecting the control of agglomeration externalities [22]. However, the impact of agglomeration externalities on firm location behavior, especially that of polluting plants, cannot be ignored [24,25,26]. Zeng and Zhao [23] proposed a theoretical model of agglomeration effects in the context of ER and carried out strict mathematical derivation. They proved that manufacturing agglomeration forces can alleviate the pollution haven effect. Wagner and Timmins [22] strongly supported Zeng and Zhao’s [23] view, and they found supporting evidence in the German chemical industry. The latest related research comes from Pang et al. [13]. They explored the economic threshold of effective ER in the Chinese context. Agglomeration is seen as the corresponding product of economic growth in the regional dimension [27]. Thus, the research ideas of Pang et al. [13] are very similar to those of Zeng and Zhao [23].

Clearly, discussing the mechanism and effect of ER on the location of new polluting enterprises from the agglomeration perspective is useful for clarifying the PH and PHH. More importantly, such discussion provides a new research angle for addressing the major unresolved public policy issue of how to determine the optimal institutional arrangement for pollution control.

**Figure 1:**
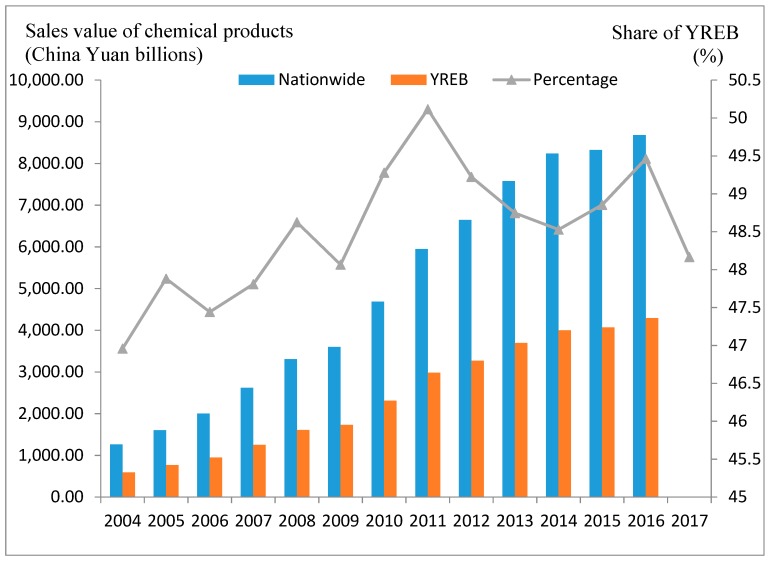
The sales value of chemical products in the YREB and its proportion in China.

The ER practices in China, the world’s largest developing country, have extensive representativeness and referential value. The strict control of chemical enterprises in the jurisdiction of the Yangtze River Economic Belt (YREB) is a typical case. In fact, the production and distribution of chemical products can be very dangerous, which has generated a widespread aversion to chemicals [28]. In particular, since the explosion at a chemical industrial park in Jiangsu Province in March 2019, the entire YREB has exercised unprecedented strict supervision over chemical firms along the river. The YREB covers nine provinces and two municipalities across the country, spanning the three gradient terrains of China. Even though development gaps between regions are obvious, most of them rely on the Yangtze River golden waterway to concentrate the development of heavy polluting industries represented by chemicals. Over the past decade or so, the total sales value of the chemical industry in the region has accounted for an average of more than 48% of the total in China (Figure 1). However, the environmental problems in the region are very serious. For example, in this region, the discharge of industrial wastewater accounts for more than 40% of that in China, and the industrial sulfur dioxide emission per unit area is 1.5–2.0 times the national average [29]. Therefore, in recent years, the central government has clearly stated that it is necessary to implement more stringent ERs in this region. Additionally, the accumulation of production factors must be guided to advantageous areas to achieve green development transformation [30]. More importantly, in recent years, the number of new chemical firms and environmental standards in different regions of the YREB has shown significant differences. These differences provided an excellent case study for us to explore how ERs affect the spatial layout of chemical firms.

This paper used a newly collected and constructed database of new chemical enterprises in 108 prefecture-level cities in the YREB from 2013 to 2018. First, we used an individual fixed-effects panel model to estimate the relationship between ERs and the location behavior of new chemical firms controlling the agglomeration. The results were used as a benchmark. Second, we constructed a regional agglomeration index as the threshold variable, and we used a fixed-effects panel threshold model to explore the agglomeration threshold of effective ER. Finally, considering the impact of spatial heterogeneity, we further divided the YREB into three regions: the upper, middle, and lower reaches of the YREB. Our results provided empirical support for Zeng and Zhao’s [23] theoretical model. They can also provide a basis for local governments to implement reasonable environmental protection policies according to the industrial agglomeration within their jurisdictions.

The rest of this study is organized as follows. Section 2 analyzes the mechanism of the impact of ER on the location behavior of new polluting firms and proposes relevant hypotheses. Section 3 presents the data and descriptive analysis, introducing the features of the new database we built. Section 4 briefly introduces the relevant models, reports the regression results in detail, and evaluates the hypotheses proposed in Section 2. Section 5 summarizes the research results and policy implications of this paper and discusses possible extensions in future research.

## 2. Mechanism Analysis and Hypothesis Development

In general, environmental problems in regions with high industrial concentrations are more prominent, and thus, these regions are faced with more stringent ERs. In fact, people’s perceptions of environmental pollution are generally more closely associated with the industrial agglomeration than with individual firms [31]. Furthermore, these perceptions will eventually evolve into formal or informal environmental governance policy [19], which will increase compliance costs for firms. However, higher industrial agglomeration can improve the production efficiency of enterprises through knowledge spillovers, labor matching, and sharing of intermediate inputs, thus leading to higher income at the same time. Therefore, for new entrants, the final location depends on the tradeoff between these two opposite forces.

**Figure 2:**
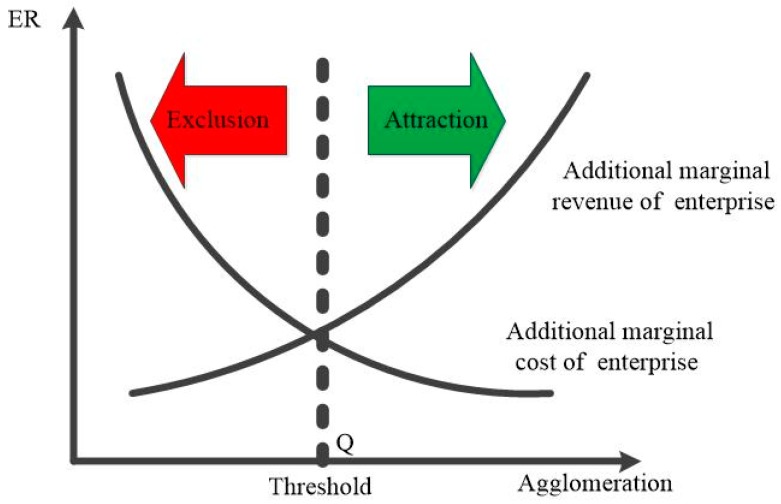
The agglomeration threshold of environmental regulation (ER): a mechanism analysis model.

As shown in Figure 2, we denoted the point of intersection between the additional marginal revenue curve and the additional marginal cost curve of enterprises as point Q. Clearly, at point Q, the incremental marginal cost of enterprises (caused by ER) is exactly equal to their incremental marginal revenue (caused by industrial agglomeration). In other words, the ER intensity and industrial agglomeration level at point Q make the additional profit obtained by new entrants exactly zero. Therefore, the region neither repels nor attracts new polluting firms. However, before the industrial agglomeration reaches the threshold (i.e., to the left of point Q), the benefits of agglomeration will not cover the cost of environmental regulation, and the new polluting firms will not find the place attractive. As a result, this region shows a repellant effect on new polluting enterprises. Conversely, when the degree of agglomeration exceeds the threshold (i.e., to the right of point Q), the region is attractive to new polluting firms.

**Figure 3:**
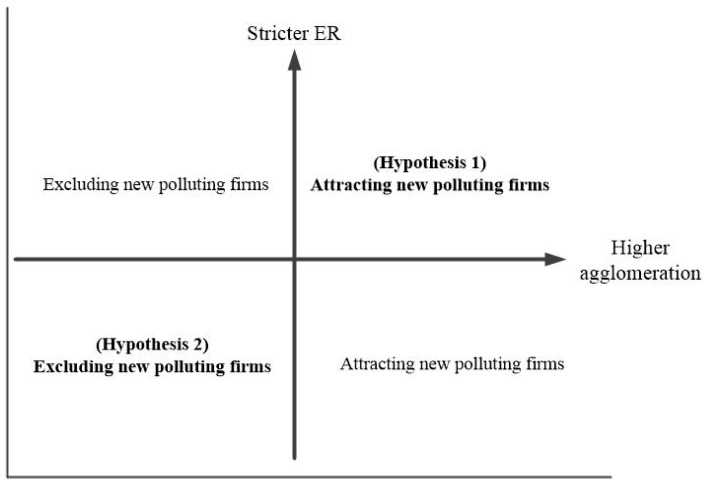
Schematic diagram of the research hypotheses.

We divided an alternative space for the site selection of new chemical enterprises into four categories (Figure 3). We then studied the influence of ER on the location behavior of new chemical firms at different industrial agglomeration levels and developed the following hypotheses.

As shown in Figure 3, different combinations of ER and industrial agglomeration divide the whole plane into four quadrants. Among these, Quadrant I represents a region with a high industrial agglomeration and strict environmental standards (“high–high” combination). Analogously, Quadrant II is the “low–high” combination, Quadrant III is the “low–low” combination, and Quadrant IV is the “high–low” combination.

According to the PHH, strict ER standards will increase barriers to entry for new chemical firms through “cost effects”. In contrast, according to the PH, high level of industrial agglomeration and strict ER standards often lead to an “innovation compensation effect” [32,33], thereby increasing the attractiveness of the region to new firms. Given the actual situation in the YREB regions, we conjectured that the YREB downstream region may be categorized into the “high–high” combination of Quadrant I. Furthermore, we proposed

**Hypothesis** **1.** *In Quadrant I, the agglomeration level exceeds the agglomeration threshold of effective ER, and the regions that meet this condition are attractive to new chemical firms.*

In addition, the regions represented by Quadrant III have loose ER standards but low industrial agglomeration. Loose environmental regulatory standards can reduce related compliance costs for businesses, attracting polluting plants. However, the lower level of industrial agglomeration means that the firms in the region cannot enjoy the positive externalities produced by the agglomeration effect, which is not conducive for new firms to invest in and set up factories. In fact, the actual development of the YREB middle and upstream regions is more consistent with the “low–low” combination of the Quadrant III. Thus, this paper proposed

**Hypothesis** **2.** *In Quadrant III, the level of agglomeration does not reach the threshold of effective ER and the regions that meet the characteristics of this quadrant are not attractive to newly established chemical enterprises.*

Finally, the impact of the regions represented by Quadrants II and IV on the location of new polluting firms is obvious. Specifically, the “low–high” combination of AG and ER in quadrant II has a repellant effect on new chemical firms. Conversely, the “high–low” combination of AG and ER in quadrant IV significantly increases the incentives for new polluting plants to set up firms in these regions. This is in line with the above analysis and general economic intuition.

## 3. Data Source and the Dynamics of the Location of New Chemical Firms in the YREB

### 3.1. Data Source

#### 3.1.1. Dependent Variable

Y was the dependent variable, representing the number of chemical companies newly registered in the YREB each year with registered capital of more than RMB 1. The reason is that since 2014, China has converted the registered capital of industrial enterprises from paid-in registration to subscription registration. The data were derived from the database of newly established chemical firms in the YREB established in this paper, namely, the YREB Annual Statistics of New Chemical Firms (ASNCF) database. We retrieved the relevant raw data mainly from official Chinese websites such as the “National Enterprise Credit Information Publicity System” and “Credit China”, and some data were supplemented by looking up the “Global Enterprise Database from Wind Information”. To the best of our knowledge, the ASNCF database established in this paper is the only, latest, and most complete enterprise database in China. It is an effective supplement and extension to the “China Annual Survey of Industrial Firms” (ASIF). The ASIF has long been the core database used by scholars from all over the world to study the behavior of Chinese enterprises. However, the database itself has some defects [34] and has not been updated since 2013.

#### 3.1.2. Core Explanatory Variable and Threshold Variable

The core explanatory variable in this paper is the strictness of ER, and the industrial SO_2_ removal rate was used as a proxy variable. The calculation method used was the amount of industrial SO_2_ removal/the total amount of industrial SO_2_ production. The reasons for this were as follows: First, SO_2_ is one of China’s major pollutants and is a key target of industrial pollution control [4,35]. This makes the collection of industrial SO_2_ emissions data relatively easy and accurate. Second, using the SO_2_ removal rate as a proxy variable for ER makes it possible to avoid overly harsh assumptions. For example, we do not need to assume a particular relationship between environmental policy and environmental performance, nor do we have to assume that the pollution abatement cost is an exogenous factor in the decision making of firm location [22]. Moreover, because of good comparability [14], this approach has been accepted by many researchers [18,22,36,37]. We used the degree of industrial agglomeration as the threshold variable. Considering the possibility of industrial data at the prefecture-level city scale, this paper drew on the research methods of O’Donoghue and Gleave [38] and He et al. [39], and we used the location quotient of the employed population in an industry to calculate the industrial agglomeration. Finally, we tested the robustness of the empirical results, and took the ratio of wastewater centrally treated as another proxy variable of ER.

#### 3.1.3. Control Variables

We selected market potential, traffic conditions, and labor costs as controlled variables. We followed the empirical literature [40] and took GDP as a proxy for market size, we used highway density as a proxy for transportation infrastructure [25], and we included the average wage of employed labor as a proxy variable for labor costs. According to the dartboard theory [41], the dependent variable may have been affected by area size. Therefore, to control this variable, we included the land area of a city [22], which is recorded in Table 1, which details the calculation formulas, sources, and statistical characteristics of all variables above.

### 3.2. The Dynamics of the Location of New Chemical Firms in the YREB

**Figure 4:**
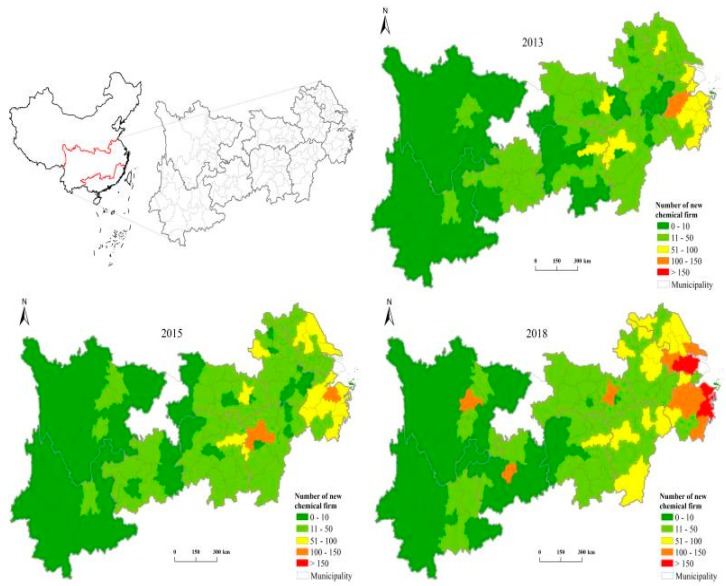
Location dynamics of new chemical firms in prefecture-level cities in the YREB.

Based on the ASNCF database, we used ArcGIS to depict the characteristics of the spatiotemporal evolution of the location distribution of new chemical enterprises in the YREB (Figure 4). We chose three representative time-points of 2013, 2015, and 2018 to perform a detailed analysis. Overall, the number of new chemical enterprises is increasing. In 2018, the number of new chemical firms increased by approximately 2000, or more than 66% compared with that in 2015. Second, in terms of spatial distribution, new chemical firms have been continuously concentrated in the downstream coastal cities and the core cities in the middle and upper reaches in the past 6 years. In other words, the location of new chemical enterprises has a typical distribution feature of “center–periphery”. The core regions mainly include the downstream coastal cities, the middle and upstream provincial capitals, and economically developed prefecture-level cities. This result does not support the conclusion of many scholars that the ERs in different regions of China lead to the transfer of a large number of polluting firms from the east to the west [42].

In fact, compared with “peripheral regions”, “core regions” generally have higher environmental standards and higher industrial agglomeration levels. The core regions showed a stronger attraction for new chemical firms. However, in the midstream and upstream regions, not all provincial capitals and developed regions were significantly appealing to new chemical firms. This result indicates that the agglomeration effect plays an important role in the influence of ER on the site selection of new polluting enterprises. It also suggests that the effect of environmental regulation is not linear. Thus, the results initially supported our hypothesis that there may be a threshold for agglomeration.

## 4. Model Specifications and Empirical Analysis

### 4.1. Model Specifications

Coughlin and Segev [43] and List and Mechone [44] converted the enterprise location selection problem into a problem of distribution of the number of new enterprises in spatial location. In this way, they solved the problem of firm location selection on a small spatial scale and with more spatial units. Since short panel data (t = 6, n = 108) were used in this paper, the influence of the time factor was controllable. Therefore, we adopted an individual fixed-effects model. Additionally, because there is often a time lag for explanatory variables to influence the location of new chemical firms, and there may be endogenous problems between some explanatory variables and explained variables, we lagged all explanatory variables by one stage. Specifically, we used the panel data individual fixed-effects model to carry out regression analysis with Equation (1), and preliminarily examined the role of industrial agglomeration in ER affecting the location selection of new chemical enterprises.

(1) $$Y_{it}=\alpha_{i}+\beta_{1}ER_{it-1}+\beta_{2}AG_{it-1}+\beta_{3}ER_{it-1}^{2}+\beta_{4}ER_{it-1}*AG_{it-1}+\phi CV_{it-1}+\nu_{i}+\varepsilon_{it}$$

(2) $$CV_{it-1}=(\ln(MP_{it-1}),\ln(WL_{it-1}),TD_{it-1},S_{i})$$

where *CV_it-1_* is a set of control variables; *ν_i_* is the prefecture-level city fixed effect, which is used to control the effects of omitted variables; *ε_it_* is an i.i.d error term; and *β_i_* and *ϕ* are parameters to be estimated.

We then used Hansen’s [45] “threshold regression” panel model to explore the agglomeration threshold of effective ER. The relevant regression equation was as follows.

(3) $$Y_{it}=\alpha_{i}+\lambda_{1}ER_{it-1}(AG\leq \gamma_{1})+\lambda_{2}ER_{it-1}(\gamma_{1}<AG\leq \gamma_{2})+\ldots +\lambda_{n+1}ER_{n+1}(\gamma_{n}<AG)+\varphi CV_{it-1}+\nu_{i}+\varepsilon_{it}$$

### 4.2. Empirical Analysis

#### 4.2.1. Individual Fixed-Effects Panel Regression

To observe the influence of ER on the location behavior of new chemical enterprises under different agglomeration conditions, we first conducted a quantitative analysis that did not include the interaction term (AG*ER) of agglomeration (AG) and environmental regulation (ER). AG*ER was then added to the regression model, and the differences between the two regression results were compared. Additionally, we separately tested the upper, middle, and lower reaches of the YREB. The results proved that there is spatial heterogeneity in the impact of ER on the location behavior of new chemical plants under different agglomeration conditions. Furthermore, based on the PH, we added the square term of ER to the model. The empirical results are shown in Table 2.

First, from Model 1 to 4, we found that the effect of ER on the location behavior of new chemical enterprises was U-shaped. Without the interaction term (ER*AG), higher agglomeration level had a large and significant attraction effect on newly established chemical enterprises in the middle and lower reaches. However, in the upstream region, we observed an opposite exclusion effect. Control variables such as labor costs and traffic density also showed significant spatial differences. These findings confirmed the necessity of dividing the YREB into different regions for separate analysis.

Second, after adding ER*AG to the regression, we found that higher level of agglomeration significantly changed the effect of ER on the location of new chemical plants throughout the YREB and its downstream regions. Not only did the negative influence of ER on new chemical firms become smaller, but the regression coefficient of ER changed from significant to nonsignificant as well. Thus, the model preliminarily confirmed that the effect of ER on the location of new chemical firms is affected by agglomeration, and that the effect has a threshold feature. However, the agglomeration in the midstream region did not change the repellant effect of ER on new chemical enterprises. This may have been related to the fact that the level of agglomeration in this region has not yet reached the threshold value of qualitative change. In particular, we found that except for the upstream regions, the regression coefficients of S in other regions were significantly larger than the number of new chemical enterprises in the unit land area of the region. This result contradicted the dartboard theory. Furthermore, it indicated that except in the upstream regions, newly established chemical enterprises in the YREB exhibit the agglomeration effect.

In summary, the regression results of the panel data individual fixed-effects model preliminarily confirmed that the effect of ER on the location behavior of new chemical firms is affected by agglomeration. Moreover, this effect has a threshold feature. That is, in the YREB, the effect of ER on the location of new chemical enterprises is complex and nonlinear, and there may be an agglomeration threshold for effective ER.

#### 4.2.2. Fixed-Effects Panel Threshold Regression

We validated and determined the agglomeration threshold for effective ER based on the fixed-effects panel threshold models. We conducted the threshold effect test and the threshold values using an estimate of Equation (3) with 300 bootstrap iterations. The test results are shown in Table 3.

First, for the entire YREB, at a level of significance of no more than 10%, a single threshold effect was significant, with an AG threshold of 1.994. This result was not affected by the time fixed effect and had strong robustness. Specifically, in 2018, Suzhou, Wuxi, Jiaxing, Lu’an, and Fuyang were the only five prefecture-level cities in the YREB with a degree of agglomeration greater than 1.944. The agglomeration values of the other 103 prefecture-level cities were all below this threshold.

Second, there were significant spatial differences in the agglomeration threshold variable, and the threshold value decreased successively from the downstream to the upstream. These findings were in line with the industrial agglomeration level and ER intensity in different regions. At a level of significance of no more than 10%, there was a significant double threshold effect in the lower reaches of the YREB, with agglomeration thresholds of 1.804 and 2.091. Taking 2018 as an example, only Suzhou exceeded the degree of industrial agglomeration of 2.091. The regions where the industrial agglomeration level was between the first and second thresholds included Jiaxing and Wuxi. The industrial agglomeration values of the other 21 prefecture-level cities are all below 1.804. Similarly, there was a significant single threshold effect in the middle reaches, with agglomeration threshold of 0.786. In 2018, 33 prefecture-level cities in the middle reaches exceeded this threshold. There was no significant threshold effect in the upper reaches.

In general, the industrial agglomeration threshold was relatively small in regions with lax ERs. The reason may have been that the additional marginal costs of lax ER were small; to offset these costs, only small additional marginal benefits from low level of industrial agglomeration were needed.

Table 4 summarizes the estimated results of the panel threshold model. For each model, due to the size of the threshold variable, multiple coefficients of the ER variables were nonlinear.

First, from the perspective of the whole YREB, the influence of ER on the location of new chemical plants was dynamic. The effect was closely related to the regional industrial agglomeration level. Specifically, after introducing the threshold, when AG < 1.944, there was a negative impact of ER on the number of new chemical enterprises. However, the negative effect was small and nonsignificant. In contrast, when AG exceeded this threshold, ER exhibited a significant positive effect on the number of new chemical enterprises, and the regression coefficient increased remarkably. That is, when AG > 1.944, for every 1% increase in the industrial SO_2_ removal rate in the YREB, the number of newly registered chemical enterprises in this region increased by an average of 0.951.

Second, in the lower reaches of the YREB, the impact of ER on the location of new chemical firms conformed to the Porter hypothesis. Under high industrial agglomeration and strict environmental standards, the benefits gained by enterprises from the agglomeration effect could cover or even exceed the cost increases caused by pollution control. As shown in Figure 2, the “innovation compensation effect” of ER on polluting firms was realized. **This result confirmed Hypothesis 1.** Specifically, when AG < 1.804, ER had a certain inhibitory but not significant effect on new chemical firms. With an increase in agglomeration level, when 1.804 < AG < 2.091, the impact of ER on new chemical firms underwent a qualitative change from exclusion to attraction. When AG > 2.091, ER exhibited a very strong and significant attraction effect on new chemical firms.

Third, in the middle reaches of the YREB, the pollution haven hypothesis was more explanatory. Overall, ER has always had a repellent effect on new chemical enterprises in this region. However, with a change in AG, the magnitude and significance of the exclusion effect changed rapidly. When AG < 0.786, ER had a very significant inhibitory effect on new chemical enterprises. However, once AG exceeded 0.786, the effect was reduced by tens of times and is no longer significant.

Finally, in the upper reaches of the YREB, ER had a small and nonsignificant negative effect on new chemical firms no matter the agglomeration level. The economic level of the upper reaches, especially the overall development of the industrial economy, is underdeveloped, and the level of industrial agglomeration is low. Additionally, the region has a high environmental capacity. Although the local government has adopted loose environmental policies, the number of chemical firms that have settled here is still limited. **This finding confirmed Hypothesis 2.** The main reason is that regions with low industrial agglomeration produce limited positive externalities. Therefore, it is difficult or even impossible for enterprises in the region to obtain additional marginal benefits, rendering the region unattractive for new entrants.

#### 4.2.3. Robustness Test

To ensure the robustness of the empirical results, the paper tested the robustness by changing the proxy variable of ER (Table 5 and Table 6). Since wastewater is another major pollutant in chemical industry, we used the ratio of wastewater centralized treated to measure the strength of ER. The results showed no obvious difference from the results in Table 3 and Table 4. The regression coefficients of the explanatory variables and control variables were stable in terms of significance and value. Therefore, it was concluded that the empirical results in this study were robust and reliable.

## 5. Conclusions

This study analyzed the mechanism of the impact of ER on the location behavior of newly built polluting firms. It then took the number of new chemical enterprises in the YREB as an example to verify and estimate the agglomeration threshold of effective ER. The results showed that there is a single agglomeration threshold for the whole YREB region that represents the turning point of ER from excluding to attracting new chemical enterprises. Additionally, there are two agglomeration thresholds in the lower reaches. If agglomeration reaches the lower threshold, the effect of ER changes from repulsion to nonsignificant attraction. Once above the upper threshold, the attraction effect becomes large and significant. Furthermore, there is a single agglomeration threshold in the middle reaches. When agglomeration level exceeds the threshold, the repellant effect of ER is no longer significant. In the upper reaches, we found no valid threshold and ER always exhibited a small and nonsignificant exclusion effect.

The marginal contribution of this paper lies in the following two aspects. First, this article enriches the perspectives and methods of related research. Based on the threshold effect model, we effectively identified the different effects of ER on the spatial–temporal pattern of newly-built chemical firms under different degrees of industrial agglomeration. It responded to the current academic debate about the effects of ER on the spatial–temporal pattern of polluting industries. We found the prerequisites that make the Porter hypothesis or pollution haven hypothesis valid. In fact, we found that in the lower reaches of the YREB, the impact of ER on the spatial–temporal pattern of polluting enterprises is in line with Porter hypothesis. In the middle and upper reaches, the impact of environmental regulation on the spatial–temporal pattern of polluting firms conforms to the pollution haven hypothesis. This paper also provides empirical support for Zeng and Zhao’s [23] argument. More importantly, the chemical industry is representative of polluting industries more generally, so the analytical framework and methods of this paper are also applicable to the related research on other polluting industries.

Second, this paper provides a basis and reference for the Chinese government to formulate differentiated environmental protection policies. Our research results indicated that, under different agglomeration conditions in different regions of the YREB, the impact of ER on the number of new chemical firms is complex and nonlinear. However, in China, environmental policies are formulated without considering regional differences. In the whole country, environmental policies are generally formulated and implemented uniformly, and the same standards are implemented on a region-wide basis [46,47]. Clearly, such unified environmental standards sometimes lead to less stringent regulations in the lower reaches of the YREB, resulting in governance that is too weak. Conversely, for the upper reaches, these regulations may be too strict, leading to difficulties in implementation and administration. Therefore, the local governments should combine their own industrial agglomeration level and the effective agglomeration threshold to determine the best environmental institutional arrangement in line with local suitability.

Future work may build on this study in several ways. On the one hand, variables such as firm size could be considered. For example, future research should distinguish the scale of new chemical enterprises and further study the agglomeration threshold of effective ER at different scales. On the other hand, future research should pay more attention to improving the measurement methods for core explanatory variables (such as environmental regulation, industrial agglomeration, etc.) and strive to make the measurement of related variables more accurate. In addition, research based on smaller spatial scales will make the results more accurate, and the recommendations derived from them will be more targeted and operable. Therefore, research on smaller spatial scales is also an important topic for future research, especially for empirical testing.

## Figures and Tables

**Table 1:** Calculation method of variables and its basic statistical characteristics.

Variable Set	Variables	Variable Definition	Data Sources	Basic Statistics				
				Mean	Std.	Min	Max	Obs.
Dependent variable	Number of newly registered chemical firms, Y	The number of chemical firms in each prefecture-level city	★☆＊	34.32	42.55	0	420	648
Core explanatory variables	Environmental regulation, ER	Removal rate of SO_2_ (%)	●	59.72	26.56	0	99.52	648
	Environmental regulation, ER’	Ratio of wastewater centralized treated (%)	●	84.64	11.77	23.47	100	648
	Industrial agglomeration, AG	Location quotient of the employed population in industry (dimensionless)	●▲	0.87	0.43	0.03	3.15	648
Controlled variables	Market potential, MP	Regional gross national product (2000 constant prices, CNY billions)	●	135.97	146.25	12.67	890.38	648
	Wage level, WL	Average wage of employees on the job (CPI_2012_ = 100, CNY hundreds)	●	499.61	116.54	260.94	922.99	648
	Traffic density, TD	Length of highway/land area (km/km^2^)	◎	1.26	0.40	0.16	2.63	648
	Land area of administrative unit, S	Land area of prefecture-level city (km^2^)	◎	12,595	7817	1459	44,266	108

★ National Enterprise Credit Information Publicity System [http://www.gsxt.gov.cn/] (accessed on 2 Jan 2020); ☆ Credit China [https://www.creditchina.gov.cn/] (accessed on 2 Jan 2020); ＊ Global Enterprise Database from Wind Information; ● National Bureau of Statistics of China. China City Statistical Yearbook (2013–2018); ▲ National Bureau of Statistics of China. Input-output tables of China (2015); ◎ Statistical yearbook of each prefecture-level city in the Yangtze River Economic Belt (2013–2018).

**Table 2:** Estimation results of the individual fixed-effects models.

ln(Y)	Whole YREB Model 1		Lower Reaches Model 2		Middle Reaches Model 3		Upper Reaches Model 4	
ER	−1.512 ** (−2.41)	−0.778 (−1.27)	−2.805 *** (−4.75)	−0.604 (−1.56)	−0.115 (−1.32)	−0.109 (−0.81)	−0.583 ** (−2.24)	−0.501 ** (−1.96)
*ER*2	0.012 *** (6.03)	0.010 *** (5.44)	0.027 *** (3.82)	0.021 *** (2.72)	0.004 * (1.86)	0.003 * (1.75)	0.005 * (1.78)	0.004 (1.52)
AG	6.581 (1.26)	−25.245 * (−2.03)	46.719 * (1.89)	−63.283 (−0.70)	7.064 ** (2.42)	0.129 (0.02)	−1.552 (−0.22)	−18.782 (−1.31)
ER*AG		0.486 * (1.69)		1.434 ** (2.15)		−0.118 (−1.19)		0.275 (1.10)
Ln(MP)	46.278 *** (2.68)	53.415 *** (3.11)	117.553 (1.22)	132.095 (1.43)	47.539 *** (3.81)	55.346 *** (4.30)	12.988 (0.91)	15.063 (1.04)
Ln(WL)	12.565 (0.93)	7.503 (0.58)	139.702 (1.47)	129.092 (1.41)	−12.528 (−1.26)	−21.718 ** (−2.05)	5.374 (0.55)	5.363 (0.56)
TD	−5.869 (−0.48)	−4.009 (−0.34)	−180.084 (−1.41)	−177.171 (−1.45)	12.770 * (1.68)	12.789 * (1.66)	13.651 (0.90)	13.670 (0.92)
S	0.003 (0.96)	0.002 (0.91)	0.011 (1.31)	0.009 (1.27)	0.002 (1.06)	0.002 (1.01)	0.001 (0.86)	0.000 (0.79)
Constant	−436.9 *** (−5.76)	−421.8 *** (−5.83)	−2066.4 *** (−4.91)	−2019.1 *** (−4.81)	−206.5 *** (−3.28)	−166.5 *** (−2.69)	−103.2 (−1.43)	−108.6 (−1.49)
Individual-FE	Y	Y	Y	Y	Y	Y	Y	Y
*R* ^2^	0.671	0.677	0.567	0.591	0.807	0.811	0.631	0.634
F-stat.	17.65	15.57	7.36	6.73	17.71	12.08	6.56	5.33
Obs.	648	648	144	144	312	312	192	192

Notes: T values are in parentheses below the coefficients; * *p* < 10%, ** *p* < 5%, *** *p* < 1%.

**Table 3:** Results of the threshold effect tests (bootstrap = 300).

The whole YREB	Threshold	F-stat.	Prob.	Crit10	Crit5	Crit1	Threshold Estimator
	Single	54.66	0.03	24.998	33.403	66.726	Th-1 1.944
	Double	8.37	0.61	52.323	72.411	135.36	Th-21 1.944, Th-22 1.570
Lower reaches	Single	30.92	0.01	18.372	25.647	29.902	Th-1 2.091
	Double	25.69	0.09	23.990	59.034	112.761	Th-21 2.091, Th-22 1.804
	Triple	4.98	0.61	20.673	30.792	65.252	Th-31 2.091, Th-32 1.804, Th-33 2.033
Middle reaches	Single	8.32	0.00	5.570	6.496	7.833	Th-1 0.786
	Double	5.55	0.21	6.858	7.438	9.064	Th-21 0.803, Th-22 0.515
Upper reaches	Single	26.05	0.23	40.871	50.976	63.045	Th-1 0.506

**Table 4:** Estimation results of the threshold models.

ln(Y)	The Whole YREB Model 5	Lower Reaches Model 6	Middle Reaches Model 7	Upper Reaches Model 8
Ln(MP)	74.579 *** (3.95)	179.905 ** (2.08)	22.339 *** (11.89)	12.309 (0.86)
Ln(WL)	−10.475 (−0.65)	68.334 (0.83)	2.645 (0.38)	−0.296 (−0.02)
TD	−0.054 (−0.00)	−172.372 (−1.32)	−5.285 * (−1.62)	18.852 ** (1.95)
S	0.003 (1.02)	0.012 (1.33)	0.002 (1.14)	0.001 (0.93)
Constant	−373.1 *** (−4.09)	−1815.9 *** (−4.14)	−139.6 ** (−1.90)	−93.8 (−1.24)
ER(AG≤1.944)	−0.149 (−1.44)			
ER (AG>1.944)	0.951 *** (5.73)			
ER (AG≤1.804)		−0.212 (-0.43)		
ER(1.804<AG≤2.091)		0.802 (1.45)		
ER (2.091<AG)		2.066 *** (3.00)		
ER (AG≤0.786)			−0.117 ** (-2.39)	
ER (AG>0.786)			−0.016 (-0.33)	
ER (AG≤0.506)				−0.411 (−0.35)
ER (AG>0.506)				−0.035 (−0.33)
Individual-FE	Y	Y	Y	Y
*R* ^2^	0.70	0.72	0.66	0.51
Obs.	648	144	312	192

Notes: T values are in parentheses to the right of the coefficients; * *p* < 10%, ** *p* < 5%, *** *p* < 1% (same below).

**Table 5:** Results of the threshold effect tests (bootstrap = 300).

The whole YREB	Threshold	F-stat.	Prob.	Crit10	Crit5	Crit1	Threshold Estimator
	Single	18.71	0.07	17.605	22.159	30.742	Th-1 1.938
	Double	22.06	0.09	19.257	24.793	24.793	Th-21 1.938, Th-22 1.491
Lower reaches	Single	31.48	0.05	19.920	30.06	49.87	Th-1 2.090
	Double	29.55	0.08	15.264	31.790	43.461	Th-21 2.090, Th-22 1.803
	Triple	5.55	0.45	18.541	27.946	51.716	Th-31 2.090, Th-32 1.803, Th-33 2.027
Middle reaches	Single	9.67	0.02	5.121	7.358	11.084	Th-1 0.806
	Double	4.33	0.22	6.328	9.276	13.241	Th-21 0.806, Th-22 0.537
Upper reaches	Single	19.42	0.13	23.949	37.781	86.293	Th-1 0.505

**Table 6:** Estimation results of the threshold models.

ln(Y)	The Whole YREB Model 9	Lower Reaches Model 10	Middle Reaches Model 11	Upper Reaches Model 12
Ln(MP)	74.102 *** (3.68)	178.973 ** (2.15)	21.956 *** (12.04)	13.637 (1.04)
Ln(WL)	−10.625 (−0.69)	67.925 (0.82)	2.604 (0.40)	−0.202 (−0.03)
TD	−0.052 (−0.04)	−171.004 (−1.31)	−5.003 * (−1.71)	17.394 ** (1.96)
S	0.003 (1.02)	0.012 (1.33)	0.002 (1.15)	0.001 (0.93)
Constant	−381.0 *** (−3.96)	−1913.1 *** (−3.72)	−113.8 ** (−2.15)	−99.5 (−1.47)
ER’(AG ≤ 1.938)	−0.152 (−1.36)			
ER’ (AG > 1.938)	0.972 *** (4.11)			
ER’ (AG ≤ 1.803)		−0.207 (−0.46)		
ER’(1.803 < AG ≤ 2.090)		0.789 (1.52)		
ER’ (2.091 < AG)		2.004 *** (2.81)		
ER’ (AG ≤ 0.806)			−0.125 ** (−2.16)	
ER’ (AG > 0.806)			−0.013 (−0.40)	
ER’ (AG ≤ 0.505)				−0.012 (−0.84)
ER’ (AG > 0.505)				−0.037 (−0.10)
Individual-FE	Y	Y	Y	Y
*R* ^2^	0.67	0.69	0.60	0.51
Obs.	648	144	312	192
